# Seeding and propagation of Tau from the retina to the brain in TAU58 mice

**DOI:** 10.1002/alz.087453

**Published:** 2025-01-09

**Authors:** Grzegorz Walkiewicz, Alicja Ronisz, Simona Ospitalieri, Sandra O. Tomé, Rik Vandenberghe, Lies De Groef, Dietmar Rudolf Thal

**Affiliations:** ^1^ Leuven Brain Institute, Leuven Belgium; ^2^ Laboratory of Neuropathology, KU Leuven, Leuven Belgium; ^3^ Laboratory for Neuropathology, KU Leuven, Leuven Belgium; ^4^ UZ Leuven, Leuven Belgium; ^5^ Laboratory for Cognitive Neurology, KU Leuven, Leuven Belgium; ^6^ Department of Neurosciences, Faculty of Medicine, KU Leuven, Leuven Belgium; ^7^ Cellular Communication and Neurodegeneration Research Group, Leuven Brain Institute, KU Leuven, Leuven Belgium

## Abstract

**Background:**

The retina, an integral part of the central nervous system, can exhibit protein accumulation (Aβ and pTau) associated with neurodegenerative diseases such as Alzheimer’s disease (AD). Biochemical analysis revealed the existence of a distinct primary retinal tauopathy (PReT), differing from AD and primary age‐related tauopathy (PART) brain lysates, suggesting it as a potential precursor for AD tauopathy with possible diagnostic value. However, it remains unclear whether retinal pTau pathology can spread from the eye into the brain.

**Method:**

Brain lysates from human autopsy cases (PART and AD) from UZ Leuven biobank, Belgium, were homogenized and tau protein were extracted. Subretinal injections of brain homogenates, as well as phosphate‐buffered saline as negative control, were performed in two‐month‐old TAU58 mice (n=10/group) overexpressing human mutant 4‐repeat tau (P301S). Thirty‐three non‐injected TAU58 mice at various ages (6, 9, 12, 15 months) were used to examine age‐related retinal pTau pathology and neuronal loss. Paraffin‐embedded tissue sections were used for anti‐tau (AT8), anti‐RBPMS, and anti‐NeuN immunostainings, and Gallyas‐staining was used to visualize fibrillar tau pathology. Quantification of neuronal density and pTau pathology in brain samples and retinas were performed with QuPath software.

**Result:**

The retinal changes associated with pTau in TAU58 mice were characterized. We observed pTau threads and cytoplasmic pTau accumulation, predominantly in the central retina, increasing with age. Retinal pTau was linked to neuronal loss in both central and peripheral retinal regions. Subretinal injection of the brain homogenates accelerated retinal pTau pathology without increased ganglion cell loss. Anterograde seeding of pTau from the eye to the brain occurred in mice receiving subretinal injections from AD brain lysates, showing significant pTau pathology in the zonal layer of the superior colliculus.

**Conclusion:**

These results suggest that subretinal injection of human brain homogenates can induce tau seeding in the retina. The anterograde spreading of pTau pathology from the retina to the brain requires abundant pTau seeds, notably present in the AD brain and absent in cognitively normal PART cases.

**Funding**: SAO/FRA 2020/017

